# Potential Toxic Elements and Their Carcinogenic and Non-Carcinogenic Risk Assessment in Some Commercially Important Fish Species from a Ramsar Site

**DOI:** 10.3390/biology12081072

**Published:** 2023-07-31

**Authors:** Mohammad Belal Hossain, Md Moudud Ahmed, Yeasmin Nahar Jolly, As-Ad Ujjaman Nur, Salma Sultana, Shirin Akter, Jimmy Yu, Bilal Ahamad Paray, Takaomi Arai

**Affiliations:** 1 School of Engineering and Built Environment, Griffith University, Brisbane, QLD 4111, Australia; 2 Department of Fisheries and Marine Science, Noakhali Science and Technology University, Noakhali 3814, Bangladesh; 3 Atmospheric and Environmental Chemistry Laboratory, Chemistry Division, Bangladesh Atomic Energy Centre, Dhaka 1000, Bangladesh; 4 Department of Zoology, College of Science, King Saud University, P.O. Box 2455, Riyadh 11451, Saudi Arabia; 5 Environmental and Life Sciences Programme, Faculty of Science, Universiti Brunei Darussalam, Jalan Tungku Link, Gadong BE1410, Brunei

**Keywords:** potentially toxic elements (PTEs), Estimated Dietary Intake (EDI), carcinogenic risk, fish, freshwater wetland, Ecologically Critical Area (ECA)

## Abstract

**Simple Summary:**

This study investigated the contamination status and health risk of potentially toxic elements (PTEs) such as Hg, As, Pb, Fe, Zn, and Cu in four selected fish species from one of the largest wetlands in South Asia. The results showed that the degree of contamination varied among the fish species, with Fe being the most abundant PTE, followed by Zn, Cu, Pb, As, and Hg. Among the fish species, *Glossogobius giuris* had the highest Hg concentration, while *Nandus nandus* predominantly accumulated As. Health risk assessment showed the PTEs posed no substantial risks to consumers. The carcinogenic risks (CR) derived from Pb intake were also below the standard limit, further supporting the conclusion that the consumption of the studied fishes did not pose a potential health harm to consumers. Overall, the study provided evidence that, at the existing consumption rate, the analyzed fish species from the freshwater are safe to consumers.

**Abstract:**

Potentially toxic elements (PTEs) such as Hg, As, and Pb have become concentrated in the aquatic ecosystem as a result of increased human activities. However, these substances frequently have synergistic or antagonistic effects on the human body or other animals. As a result, there are concerns world-wide that commercially available food products, especially fish, may be contaminated with hazardous elements. In this study, samples of four selected fishes, Gutum (*Lepidocephalichthys guntea*), Baim (*Macrognathus pancalus*), Baila (*Glossogobius giuris*), Meni (*Nandus nandus*) were analyzed from one of the largest freshwater wetlands (designed as a Ramsar Site) in South Asia to evaluate PTEs contamination status and human health risk assessment. The result demonstrated that the degree of contamination for six PTEs decreased in the following sequences for fish: Fe > Zn > Cu > Pb > As > Hg. The edible part of *G. giuris* had the maximum value for Hg (0.42 μg/g dw), while *N. nandus* predominantly accumulated As (<0.41 μg/g dw). The estimated daily intake (EDI) values ranged from 0.003 to 1.75, which was much lower than the recommended values. The hazard index (HI), THQ, total target hazard quotient (TTHQ) scores through consuming fish followed the decreasing order of Fe > Hg > Cu > Zn > Pb. The values for each index were less than 1, indicating that there were no substantial health risks for the consumers. The carcinogenic risks (CR) derived from the intake of Pb ranged from 4.92 × 10^−8^ to 4.14 × 10^−8^ for males and 5.45 × 10^−8^ to 4.59 × 10^−8^ for females, which also did not exceed the standard limit (1.00 × 10^−6^). This study demonstrated that, under the existing consumption rate, there was no potential health harm to consumers from consuming the studied fishes. This study offers a chance to regularly check PTEs in this environment, reducing the contamination of heavy metals.

## 1. Introduction

Potentially toxic elements (PTEs) make up a cluster of harmful pollutants (e.g., As, Hg) due to their bioaccumulation, long-term retention, danger, and less or non-biodegradability in the ecosystem [1,2]. PTEs may accumulate in aquatic flora, microorganisms, and aquatic fauna in aquatic ecosystems, entering the human food chain and causing health issues [3,4]. They can infiltrate an aquatic ecosystem by anthropogenic activities or natural processes [5]. Anthropogenic activities include the indiscriminate use of fertilizers and pesticides containing toxic elements in agricultural fields, as well as mining, boating, smelting activities, as well as the disposal of untreated or only partially treated effluents [6,7,8,9]. Weathering, volcanic eruptions, and erosion are examples of natural sources of PTEs in aquatic ecosystems. 

One essential feature that makes PTEs harmful is their inability to decay spontaneously and in a non-detrimental manner [10]. They are dispersed throughout the water column after being introduced to an aquatic environment, released, or accumulated in sediments, where they are then eaten by biota [11]. They are dispersed in several aspects in aquatic habitats, including those precipitated by colloids, suspended particles, water-soluble organisms, and geological layers. These may contaminate raw water and sediment directly, causing sub-lethal effects or death in local fish populations [8]. It is feasible to use metallic bioavailability in aquatic organisms as a sign of PTE exposure because they are being accumulated in animals many-fold greater than their surroundings and can be multiplied and transported up the aquatic food chain to apex predators, including humans, via benthic and oceanic species ingestion [12]. On the contrary, PTEs in reduced amounts, such as Ni, Mn, Fe, Zn, and Cu are acknowledged as micronutrients that largely control how the body of a person operates biologically [4].

Haors, freshwater wetlands, are considered as unique ecosystems of national and international importance because of their critical economic and ecological roles [13]. The IUCN designated Tanguar Haor, one of the largest wetland ecosystems in South East Asia, as a Ramsar site because of its rich biodiversity. It supports a variety of rare and vulnerable aquatic species, including endemic ones [14]. It is a safe home for threatened waterfowl and every winter, almost 200 distinct species of migrating birds move to this area to establish a temporary habitat [14]. Consequently, this freshwater wetland gives direct subsistence to neighboring people, provides alternative income generation by fishing, and has significantly contributed to the nation’s food safety [15]. However, this Ramsar site is predominantly threatened by the overexploitation of fishery resources along with environmental pollution accelerated by its tourism industry. Along with environmental disturbances, the Tanguar wetland is disintegrating at an astounding rate [16]. Therefore, it is crucial to monitor the current pollution status of this valuable freshwater wetland. 

In 1999, the Bangladesh government designated Tanguar Haor as an Ecologically Critical Area (ECA) due to its critical state as a result of overuse of its natural resources. Several studies have been conducted on water quality, fish diversity, and livelihood conditions of the inhabitants of Tanguar Haor and its neighboring areas [13,15,17,18,19,20,21]. These studies reported about the loss of biodiversity and reduction of ecosystem services due to increased human activities. The key issues contributing to environmental pollution in this area were the usage of pesticides for farming practices, population growth, hanging latrines, burning oil, coal washing, sediment loadings, as well as other nonpoint sources [21] that release dangerous toxic components. Therefore, it was hypothesized that the area could be affected by pollutants including PTEs. However, to date, no detailed scientific research regarding the levels of contamination by PTEs or the specific sources of contamination has been conducted in this valuable ecosystem. Fish are considered as bio-indicators for PTEs contamination in aquatic ecosystems, and they play an essential role in the transfer of hazardous substances to humans. Thereby, assessing the PTE content in some representative fish species can help to track the health status of any ecosystem and consumers. Given the paucity of knowledge and common concern among fish consumers, this research aimed (i) to assess the extent of potentially toxic elements (Fe, Cu, Zn, Pb, and Hg) in the edible portion of some commercially important fishes collected from Tanguar Haor (ii) to identify possible sources of these toxic elements and the ecosystem health.

## 2. Materials and Methods

### 2.1. Study Area and Sampling Sites

**Figure 1:**
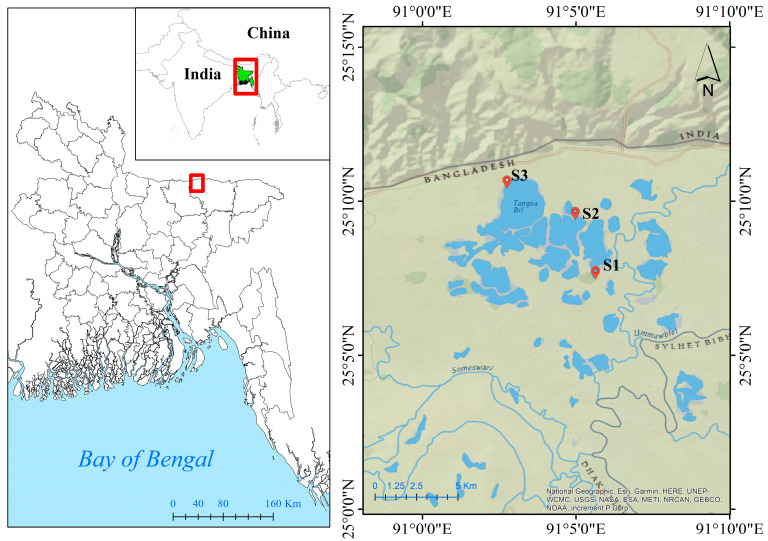
Location of study area, Tanguar Haor of Sunamganj, Bangladesh.

Tanguar Haor, known as “mother fishery”, is a nationally important wetland in Bangladesh. It is located in the Sunamganj district of Bangladesh, between 25°12′2.572″ to 25°5′47.989″ North Latitude and 90°58′49.426″ to 91°10′0.018″ East Longitude, covering an area of 160 km^2^ (Figure 1). It has a remarkably diversified biome, with 137 kinds of fish and 284 species of birds living in this wetland [18]. This haor supports the livelihoods of 88 villages and around 70,000 people in its surroundings. The Netrokona district, which is located west of the haor, is only around 2.5 km away. As sampling locations, three locations were chosen—Hathrigatha, Nainder Beel, and Lechuamara—where there was no evidence of river bank breaking and no sediment settlement. Tanguar Haor has a subtropical monsoon climate, with the three dominant seasons most likely being monsoon (July–October), summer (March–June), and winter (November–February). The summer months of April through June have temperatures between 30.9 and 33.4 °C. Temperatures in the winter, which last from October to February, range from 8.5 to 16.6 °C. The haor region receives 8000 mm of rain on average each year.

### 2.2. Sample Collection, Preparation and Metal Analyses

Fish species were collected by using seine nets from 3 different stations (S1–S3) (Figure 1). Four species of fish (*Lepidocephalichthys guntea*, *Macrognathus pancalus*, *Glassogobius giuris* and *Nandus nandus)* with different feeding habits were selected based on their commercial importance and availability. Brief morphometric data of the selected fishes are presented in Table 1. A total of 36 fish samples were analyzed for PTEs using the energy dispersive X-ray fluorescence (EDXRF). A variety of elements can be detected simultaneously using the EDXRF multi-element measuring technology. We followed the sample processing and preparation instructions provided by Rakib et al. [22] for metal analysis. Firstly, fish muscle was separated, cut into little pieces, and dried. The dried samples were then reduced to a fine powder using an agate mortar and pestle and sieved through a fine-mesh plastic sieve. A pellet maker equipped with a hydraulic press was used to create a 0.7 cm diameter and 1 mm thick pellet from 0.1 g of each powdered material by exerting 10 ton pressure for around 3 min. The pellets were then put into an X-ray excitation chamber of an EDXRF and exposed to radiation for 1000 s using a 30 mCi Cd109 annular source. A multichannel analyzer recorded the data. The computer packages PRO/QXAS (IAEA) and AXIL were used for quantitative and qualitative elemental analysis to process and evaluate the net X-ray intensities. Three pellets (Tuna-1, Tuna-2, and Tuna-3) were made from the certified reference material (CRM), and three standard tuna homogenates (IAEA-350) were utilized for calibration. By examining the spectrum of DORM-2 dogfish muscle, the accuracy and precision of the calibration curves were verified. The recoveries for the studied metals ranged from 89.9 to 96.4% (89.9% for Zn, 96.4% for Pb). 

### 2.3. Health Risks and Measuring Contamination Levels in the Study Area 

Several indices have been developed for assessing the health risk of heavy metals [4,22,23,24]. Table 2 represents the indices used for calculating contamination level and health risk in the study area. 

### 2.4. Assessing the Risk of MeHg from Fish Consumption

Hg is converted into methylmercury (MeHg) in aquatic bodies by sulfate-reducing bacteria. Therefore, measuring the amount of Hg in fish can be used to estimate exposure to MeHg. The total Hg concentration in fish is frequently employed as a proxy for MeHg exposure since practically all of the Hg found in fish and other seafood is assumed to be MeHg [25]. The following formula was used to get the MeHg (µg/kg) consumption per kilogram of body weight per week [26]:

Amount of fish ingested per week (kg/week) × Hg concentrations in fish consumed (µg/kg)(1)

kg of individual body weight.

The Provisional Tolerable Weekly Intake (PTWI) set by the Joint Expert Committee on Food Additives (JECFA, 1.6 µg/kg) and the National Research Council (NCR, 0.7 µg/kg) of the United States was used to calculate the exposure level to human health [27]. Male and female average body weights in Bangladesh were judged to be 55.2 kg and 49.8 kg, respectively, and each person consumes 62.58 g of fish every day on average.

### 2.5. Statistical Methods

Using Pearson’s correlation analysis and linear correlation analysis, the correlations between the PTEs concentrations were evaluated. The correlation analyses helped to identify the sources. Statistical Package for the Social Sciences (SPSS) version 20, free software, PAST version 3, and Sigma Plot version 12 were used for multivariate and univariate statistical analysis, including cluster analysis (CA), principal component analysis (PCA), and correlation matrix (CM).

## 3. Results and Discussion

### 3.1. Concentrations of PTEs in Fish Muscle 

**Figure 2:**
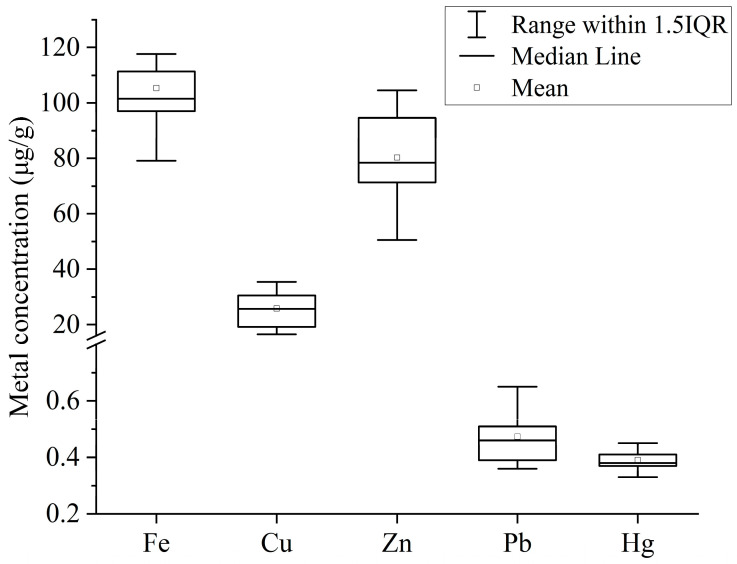
Concentrations of PTEs (μg/g) in muscle tissues of fishes from Tanguar Haor, Bangladesh.

The mean concentration of selected PTEs (Fe, Cu, Zn, Pb and Hg) in fish muscles from Tanguar Haor followed the decreasing order of Fe (105.41 ± 23.47 μg/g) > Zn (80.35 ± 18.29 μg/g) > Cu (25.9 ± 7.21 μg/g) > Pb (0.48 ± 0.11 μg/g) > Hg (0.4 ± 0.04 μg/g) (Figure 2). The results of ANOVA revealed that the concentration of PTEs differed highly significantly at 99% confidence interval (*p* < 0.01). 

The mean accumulation of PTEs in the fish species followed the decreasing order of *G. giuris* > *N. nandus* > *M. pancalus* > *L. guntea*. The maximum concentration of Zn (95.1 ± 9.5 μg/g) and Hg (0.4 ± 0.1 μg/g) was recorded in *G. giuris*, whereas Cu (27.2 ± 10 μg/g) and Pb (0.5 ± 0.2 μg/g) were documented to be higher in *L. guntea* (Table 3). In contrast, a lower concentration of Cu (23.9 ± 7.2 μg/g) and Hg (0.4 ± 0.02 μg/g) was found in *N. nandus*, whereas Zn (66.6 ± 9.7 μg/g) and Pb (0.4 ± 0.1 μg/g) were the lowest in *M. pancalus* and *G. giuris*, respectively (Table 3). Statistically, no significant differences of mean metal concentration were found among the species in case of all the analyzed PTEs (*p* > 0.05). However, in this study, pelagic fish showed a higher accumulation of PTEs than that in bottom dweller fish, which was contradictory to the previous studies [28,29]. This might be due to the feeding habits and possibly the higher load of PTEs in the water than the sediment. 

The results of the current study were contrasted with those of other Bangladeshi fish species (Table 3). Cu concentration in the analyzed fish species was found to be significantly greater than that in other species, although Pb concentration was found to be lower than in the other research [28,29,30]. Zn and Pb were found to be lower in *H. fossilis* and *Batasio batasio* than in the fish from Tanguar Haor [30,31]. The bioaccumulation of PTEs is greatly influenced by some factors such as age, trophic transfer, feeding habit, and habitat, and may even vary within the same species due to their age, body weight, and length [32]. However, the metal concentration in the studied species did not exceed the permissible limit in fish [33,34,35].

**Table 3:** PTEs concentration (µg/g) in the freshwater fish muscles from Bangladesh with guidelines.

Sampling Site	Species	Fe	Cu	Zn	Pb	Hg	Reference
Tanguar Haor, Sunamganj	*L. guntea*	88.5 ± 11.1	27.2 ± 10	73.6 ± 27.4	0.5 ± 0.12	0.4 ± 0.1	Present study
	*M. pancalus*	104.1 ± 2.8	27.1 ± 4.2	66.6 ± 9.7	0.5 ± 0.03	0.4 ± 0.03	
	*G. giuris*	109.2 ± 8.8	25.5 ± 10.3	95.1 ± 9.5	0.4 ± 0.1	0.4 ± 0.1	
	*N. nandus*	119.8 ± 45.4	23.9 ± 7.2	86 ± 13.5	0.5 ± 0.1	0.4 ± 0.02	
Mymensingh	*P. hypophthalmus*	NA	0.8 ± 0.01	NA	0.4 ± 0.2	NA	[31]
Buriganga River	*H. fossilis*	NA	8.1 ± 0.4	26.7 ± 0.1	2 ± 0.03	NA	[30]
Dhaleswari River	*Trypauchen vagina*	NA	7 ± 1.5	NA	6.9 ± 0.6	NA	[28]
Buriganga River	*Puntius ticto*	NA	11.5 ± 3.3	203.6 ± 12.9	3.1 ± 0.1	NA	[29]
	*Puntius sophore*	NA	9 ± 1.6	248.2 ± 14.6	3.2 ± 0.1	NA	
	*Puntius chola*	NA	6.9 ± 1.1	292.1 ± 19.8	2.3 ± 0.1	NA	
	*Labeo rohita*	NA	18.8 ± 2.2	251.7 ± 18.2	7 ± 0.2	NA	
	*Glossogobius giuris*	NA	5.9 ± 0.5	194.7 ± 12.6	1.8 ± 0.1	NA	
Korotoa River	*Channa punctata*	NA	0.8 ± 0.4	NA	0.5 ± 0.5	NA	[27]
	*Anabas testudineus*	NA	2.1 ± 0.7	NA	1.1 ± 0.9	NA	
	*Batasio batasio*	NA	1 ± 0.7	NA	0.3 ± 0.2	NA	
Permissible limit in fish		-	30	30	1.5	0.5	[33,34,35]

NA—Not available.

### 3.2. Sources Identification

Correlations among potential toxic elements were assessed to determine the origin and migration of PTEs [36]. The linear relationships (positive and negative) were found between the elements (Table 4). A strong positive correlation was observed between Fe and Cu (*r =* 0.79), Zn and Cu (*r =* 0.91), Hg and Cu (*r =* 0.85), Pb and Zn (*r =* 0.62), and Hg and Zn (*r =* 0.92). The correlations among Fe, Cu, Zn, Hg, and Pb indicate they originated from common sources. Islam et al. [37] reported similar metal correlations (Zn-Pb in pre-monsoon and Mn-Pb in post-monsoon seasons), which were closely associated, and showed the same origins and pathways of pollution entering the aquatic environment of the haor area.

**Figure 3:**
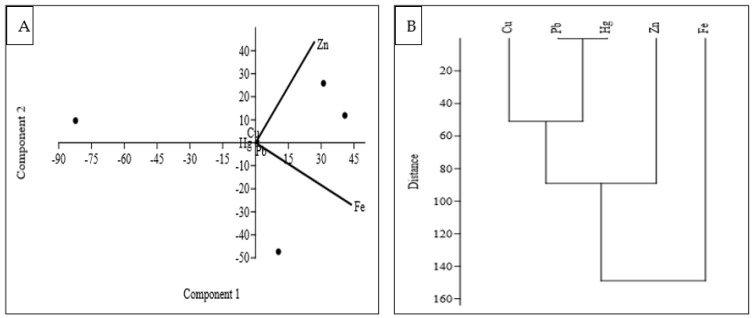
Loadings of PCA (**A**) and Dendrogram (**B**) of the studied PTEs in fish samples.

Principal Component Analysis (PCA) is used to decrease the number of variables to a smaller set of components, leading to an easier interpretation (Figure 3A). It helps to examine the data’s variability and to find patterns and reduce the dimensionality of the data. In this study, three significant principal components (PCs) that accounted for 99.97% of the data variability were found by the PCA (Table 5). In the data, 75.17% of the total variation was accounted for by PC1, the first principal component (Table 5). This component showed a very strong load for Fe and a moderate load for Zn. The high loadings of Fe and Zn on PC1 indicate that these two variables contribute significantly to the variation captured by PC1. This high proportion of variance suggests that it represents the most dominant pattern in the data. On the other hand, PC3 did not show any significant metal loading, indicating that it does not have a strong association with Fe or Zn. However, PC2, which accounted for 24.82% of the observed variation, showed a substantial positive load for Zn. This suggests that PC2 captures a distinct pattern related to Zn, but is independent of Fe. These findings provide insights into the relationships between the variables and can help in understanding the underlying factors contributing to the observed data variability.

Cluster analysis (CA) was used in this study to assemble PTEs originating from a common source [38] with all five elements grouped into two statistically significant clusters at (Dlink/Dmax) 100 < 120 (Figure 3B). Cluster 1 was made up of Fe and Zn, which had higher concentrations in the fish species studied, whereas Cluster 2 was made up of three elements (Cu, Pb, and Hg) with a lower element load in the fish. Furthermore, Pb and Hg demonstrated a significant association, with the shortest cluster distance confirming high human-caused contributions. However, our findings suggested that Fe and Zn might be derived from common sources, whereas Cu, Pb, and Hg came from anthropogenic sources. Cu, Pb, and Hg are frequently found in industrial activities, mining operations, and certain natural sources. These elements belong to the same group on the periodic table, known as Group 12 or the zinc group. Moreover, these PTEs exhibit similar chemical properties, such as the ability to form multiple oxidation states and complexes. They can enter ecosystems through various pathways and pose significant risks to environmental and human health. All of these investigations, therefore, suggested that the sources of these potentially toxic substances in the environment of Tanguar Haor could be wastewater from a sewage system, industry, or surface runoff from adjacent agricultural fields [39,40].

### 3.3. Health Implications of Fish Consumption

**Figure 4:**
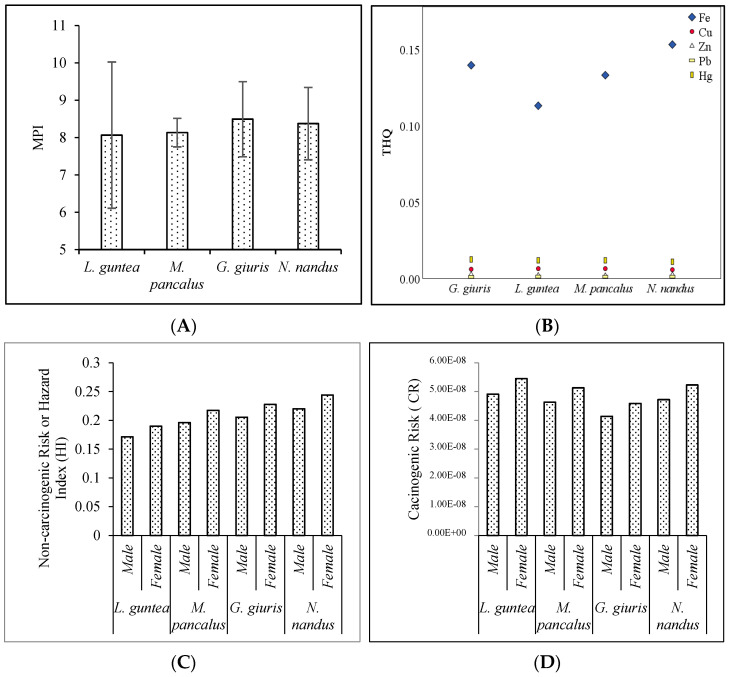
Human health-related hazard indices (**A**) MPI, (**B**) THQ, (**C**) HI, and (**D**) CR for PTE concentrations in fish samples.

The metal pollution index (MPI) is a quantitative measure used to assess the contamination level of potentially toxic elements in fish muscle. It takes into account the concentrations of various heavy metals present in the fish tissue and provides a single value that indicates the overall pollution level. The MPI values in the studied fish species from Tanguar Haor followed the decreasing order of *G. giuris > N. nandus > M. pancalus > L. guntea* (Figure 4A). Although this order indicated differences in metal accumulation and pollution levels among these fish species, the MPI values did not differ significantly (F = 0.08, *p* = 0.97) among the species. However, it has already been stated in the previous studies that feeding habits and habitat preferences greatly influence the accumulation of toxic elements [32]. *G. giuris* is a pelagic fish and has been classified as both carnivorous and herbivorous. The other three species are predominantly carnivores. Therefore, *G. giuris* acquires hazardous materials from both diets and has an increased pollution index compared to other species. The bioavailability of potentially toxic materials in aquatic organisms also differs widely throughout groups and is affected by adsorption, exposure frequency, and removal processes [25].

Figure 4B shows the target hazard quotient (THQ) of the five examined PTEs from consuming fish species. Fe > Hg > Cu > Zn > Pb were in decreasing order of the mean THQ, and the value of <1 for both adults and children suggests that adverse effects on human health might not occur. The HI or total target hazard quotient (TTHQ) scores followed the pattern of THQ. Further, average total target hazard quotient (TTHQ) values for the studied species followed the decreasing order of *N. nandus* > *G. giuris* > *M. pancalus* > *L. guntea*. However, the TTHQ values for each species were less than 1 (<1), suggesting that the fish were safe for human to consume (Figure 4C). The carcinogenic risks (CR) derived from the intake of Pb were calculated, and it was found that the values of CR ranged from 4.92 × 10^−8^ to 4.14 × 10^−8^ for males, and 5.45 × 10^−8^ to 4.59 × 10^−8^ for females (Figure 4D). However, the present findings revealed that the CR values for Pb did not exceed the standard limit (1.00 × 10^−6^), which specified that consumers of the studied species are not exposed to the cancer risk [41].

The estimated weekly intake (EWI) of PTEs through the consumption of four studied fish species is represented in Table 6. The EWI was calculated by considering that the average body weight of males, females, and children at age 10 in Bangladesh as 55.2 kg, 49.8 kg, and 34.3 kg, respectively, and a person consumes 62.58 g of fish per day. The results of EWI revealed that children are more vulnerable to PTEs than adults, which strongly supports the findings of Girolametti et al. (2022). However, the EWI values for the examined PTEs (Fe, Cu, Zn, Pb, and Hg) in the selected fish from Tanguar Haor were below the provisional tolerable weekly intake (PTWI) recommended by the WHO (1995) [35] (Table 6), indicating that the metal intake through the consumption of selected fish from Tanguar Haor may not pose any potential threats to human health.

**Figure 5:**
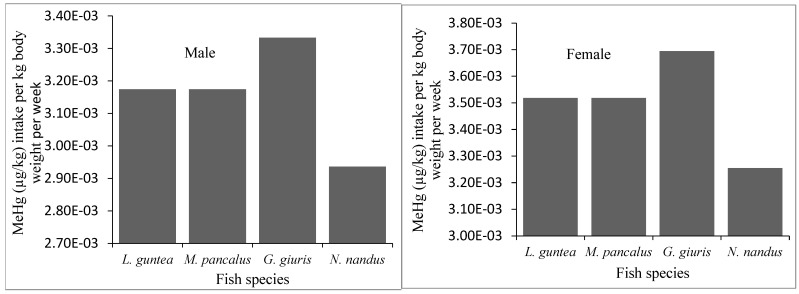
Estimated intake of MeHg by human body (adult males and females) through consumption of different fish species in the study area.

The projected intake of MeHg and the risk associated with the average consumption of fish by the local people was evaluated on the basis of the recommended provisional tolerable weekly intake (PTWI) for methylmercury, established by the Joint Expert Committee on Food Additives (JECFA, 1.6 µg/kg) and the National Research Council (NCR: 0.7 µg/kg) of the U.S [25,27]. In this study, the MeHg intake per week varied from 0.0029–0.0037 µg/kg body weight for both adult males and females (Figure 5). The weekly intake of MeHg was much below the limit of 1.6 µg/kg and 0.7 μg/kg bw/week set by the JECFA and the US-NCR, respectively. Therefore, the studied fish species were found to be safe for human consumption. 

## 4. Conclusions

This study evaluated the accumulation and health risk assessment of potentially toxic elements (Fe, Cu, Zn, Pb, and Hg) in fish for the first time from an internationally important wetland ecosystem, Tanguar Haor. PTEs were recorded from the most common food fish species. The studied fish species tend to accumulate higher levels of Fe compared to other elements like Zn, Cu, Pb, As, and Hg. Among the fish species examined, the edible part of *G. giuris* had the highest concentration of Hg at 0.416667 μg/g dw. This indicates that *G. giuris* is prone to accumulating higher levels of Hg. In contrast to *G. giuris, N. nandus* predominantly accumulates As with concentrations below 0.41 μg/g dw. This suggests that *N. nandus* has a higher affinity for arsenic accumulation compared to other elements. Health risk assessment analyses showed the potential health risks associated with PTE intake from consuming fish are relatively low. All the readings for HI and TTHQ were less than 1, indicating that there were no substantial health risks for consumers associated with these elements. The carcinogenic risks (CR) derived from the intake of lead (Pb) did not exceed the standard limit of 1.00 × 10^−6^, suggesting that the risk of cancer associated with Pb intake from fish consumption is within acceptable levels. Overall, the study indicates that the levels of PTEs in fish species were generally below recommended thresholds, resulting in minimal health risks for consumers. Nevertheless, a greater amount of PTEs in fish may cause not only human health and ecological hazards, but also the decline of the financial sector. Therefore, to secure haor-based fish production, more intensive research focusing on PTEs in natural water bodies and waste management is extremely important. Apart from this, the overexploitation of fishery resources and anthropogenic pollution sources should be taken into consideration. As a result, this research could motivate managerial and stakeholder groups to initiate appropriate action.

## Figures and Tables

**Table 1:** Biometrics data (mean ± SD) of most consumed fish from three different stations of Tanguar Haor, Bangladesh.

Sample	Habitant	N	Weight (g) ± SD	Length (cm) ± SD
Gutum (*L. guntea*)	Bottom feeder	9	5 ± 1	8.5 ± 0.5
Baim (*M. pancalus*)	Bottom feeder	9	4 ± 1	10.6 ± 0.7
Baila (*G. giuris*)	Pelagic feeder	9	4.3 ± 1.6	8.53 ± 0.85
Meni (*N. nandus*)	Bottom/column feeder	9	11.6 ± 1.6	9.3 ± 0.3

**Table 2:** Health risk indices used in the present study.

Equation	Index	Depiction and Objectives	Principle	Explanation	Pollution Degree Criteria
1.	Metal pollution index (MPI)	It is based on a long-term trend evaluation of the current situation.	MPI = C_Avg1_ × C_Avg2_ × … × C_Avgn_1/n $MPI={\left(M1\times M2\times M3\times \ldots \times M_{n}\right)}^{\frac{1}{n}}$	M1 = the concentration value of 1st metal, M2 = the value of 2nd concerned metal, and so on.	
2.	Target hazard quotient (THQ)	This is non-carcinogenic risks measured by the ratio of CDI and reference dose (RfD).	$THQ=\frac{EFr\;\times \;ED\;\times \;C\;\times \;FIR}{RfD\;\times \;BW\;\times \;AT}\times 10^{-3}\;AT=EFr\times ED$	EFr = the exposure frequency (365 days/year), ED = exposure duration (average age of human), FIR = fish ingestion rate (average adult consumption rate: 62.58 g g/person/day), C = the concentration of PTEs (mg/kg), BW is average body weight of human, AT = average exposure time (EFr × ED), RfD = oral reference doses (USEPA, 2020)	THQ < 1, no adverse health effects; for THQ ≥ 1, there could be a likelihood of possible health hazards
3.	TTHQ	The TTHQ from THQs is denoted as the total of the hazard	TTHQ=∑THQ for all metals	Summation of THQ	TTHQ > 10, high risk for its consumers.
4.	Carcinogenic Risk (CR)	It indicates an incremental probability of an individual developing cancer over a lifetime	$CR=\frac{EFr\;\times \;ED\;\times \;C\;\times \;CSF}{BW\;\times \;AT}\times 10^{-3}$	CSF = the carcinogenic slope factor set by USEPA (2010).	Risk levels for carcinogens range from 10^−4^ to 10^−6^
5.	Estimated weekly intake (EWI)	It is calculated by multiplying the respective mean concentration of the metal determined in the targeted fish samples by the weight of fish consumed by an average adult individual in Bangladesh	EWI = $\frac{DFC\;\times \;MC\;\times \;7}{BW}$	DFC = daily fish consumption (g); and MC = mean concentration of trace elements (μg/g) in fish; BW = the human body weight; 7 is the number of days in a week. (DFC = 62.58 g, BW = 55.2 kg for males, 49.8 kg for females)	The tolerable daily intake by WHO

**Table 4:** Pearson correlation analysis among PTEs in the fish samples (significance level *p* < 0.01).

	Fe	Cu	Zn	Pb	Hg
Fe	1				
Cu	0.79	1			
Zn	0.46	0.91	1		
Pb	−0.39	0.25	0.62	1	
Hg	0.49	0.85	0.92	0.44	1

**Table 5:** Principal Component Analysis of metals. Loadings, eigenvalues, explained variance of the variables for the first three PCs.

Metals	PC 1	PC 2	PC 3
Fe	0.85	−0.5	−0.01
Cu	0.03	0.02	0.1
Zn	0.5	0.85	−0.03
Pb	0.00005	0.001	0.1
Hg	0.0003	0.0004	−0.15
Variability (%)	75.17	24.82	0.001
Eigenvalues	2.61	1.01	0.63

**Table 6:** Estimated weekly intake (µg/kg body weight) of studied PTEs.

Fish Species		Fe	Cu	Zn	Pb	Hg
*L. guntea*	Male	0.702	0.216	0.584	0.004	0.003
	Female	0.779	0.239	0.647	0.004	0.004
	Child	1.130	0.347	0.939	0.007	0.005
*M. pancalus*	Male	0.826	0.215	0.53	0.004	0.003
	Female	0.916	0.238	0.587	0.004	0.004
	Child	1.330	0.346	0.852	0.006	0.005
*G. giuris*	Male	0.867	0.202	0.755	0.003	0.003
	Female	0.961	0.224	0.837	0.004	0.004
	Child	1.395	0.325	1.215	0.005	0.005
*N. nandus*	Male	0.951	0.190	0.683	0.004	0.003
	Female	1.054	0.210	0.757	0.004	0.003
	Child	1.530	0.305	1.098	0.006	0.005
PTWI (µg/person/week) [35]		392	245	490	1.75	0.35

## Data Availability

Data will be provided upon request.
